# The essential role of adenine nucleotide translocase 4 on male reproductive function in mice

**DOI:** 10.1590/1414-431X2024e13590

**Published:** 2024-05-20

**Authors:** Fengyuan Yang, Xiali Yang, Hui Zhu, Xinbo Wang, Xin Liao, Yinxu Fu, Ting Fu, Xiandan Chen, Aliaksei Sysa, Jianxin Lyu, Huaibin Zhou

**Affiliations:** 1 Key Laboratory of Laboratory Medicine, Ministry of Education, Zhejiang Provincial Key Laboratory of Medical Genetics, College of Laboratory Medicine and Life Sciences, Wenzhou Medical University, Wenzhou, China; 2 School of Laboratory Medicine and Bioengineering, Hangzhou Medical College, Hangzhou, China; 3 Key Laboratory of Biomarkers and In Vitro Diagnosis Translation of Zhejiang Province, Hangzhou, China; 4 Zhejiang Provincial People's Hospital, Affiliated People's Hospital, Hangzhou Medical College, Hangzhou, China; 5 Belarusian State University, Minsk, Belarus

**Keywords:** Adenine nucleotide translocator 4, Oxidative homeostasis, Autophagy, Male reproduction

## Abstract

Adenine nucleotide translocator 4 (*Ant4*), an ATP/ADP transporter expressed in the early phases of spermatogenesis, plays a crucial role in male fertility. While *Ant4* loss causes early arrest of meiosis and increased apoptosis of spermatogenic cells in male mice, its other potential functions in male fertility remain unexplored. Here, we utilized *Ant4* knockout mice to delineate the effects of *Ant4*-deficiency on male reproduction. Our observations demonstrated that *Ant4*-deficiency led to infertility and impaired testicular development, which was further investigated by evaluating testicular oxidative stress, autophagy, and inflammation. Specifically, the loss of *Ant4* led to an imbalance of oxidation and antioxidants. Significant ultrastructural alterations were identified in the testicular tissues of *Ant4*-deficient mice, including swelling of mitochondria, loss of cristae, and accumulation of autophagosomes. Our results also showed that autophagic flux and AKT-AMPK-mTOR signaling pathway were affected in *Ant4*-deficient mice. Moreover, *Ant4* loss increased the expression of pro-inflammatory factors. Overall, our findings underscored the importance of *Ant4* in regulating oxidative stress, autophagy, and inflammation in testicular tissues. Taken together, these insights provided a nuanced understanding of the significance of *Ant4* in testicular development.

## Introduction

Spermatogenesis is an intricate and tightly regulated process that requires coordinated support from multiple biological pathways to produce mature sperm from stem cells. Central to this complex development is the availability of adequate energy and a homeostatic environment within the seminiferous tubules of the testes (1). Oxidative stress is caused by an imbalance between reactive oxygen species (ROS) production and antioxidant defenses (2). Excess ROS can damage tissues and induce cell death, thus playing a central role in sperm production disorders (3). Elevated ROS levels can trigger inflammation, apoptosis, and autophagy through diverse pathways. Any disruption to this tightly regulated system can undermine spermatogenesis and contribute to male-related fertility problems that account for 30-50% of all infertility cases (4). This network of interdependent mechanisms creates an environment optimized for germ cell development by linking energy supply, cell death, quality control, and redox homeostasis. If any part of this system is disrupted, it can lead to a spiral of damage that culminates in reproductive dysfunction.

Autophagy, a lysosome-dependent degradation process, is essential for spermatogenesis as it regulates germ cell differentiation and removes damaged components. During autophagy, the targeted cytoplasmic contents are engulfed within autophagosomes and transported to lysosomes for breakdown and recycling. This provides a source of nutrients for driving cellular processes when energy levels are limited (5,6). In the testes, autophagy enables germ cell development under energy-restricted conditions in the seminiferous tubule and helps remove organelles that might otherwise cause oxidative stress if left unregulated (7). Previous studies have demonstrated that disrupting autophagy causes an accumulation of damaged mitochondria and abnormal germ cells, culminating in cell death and infertility (6,8). Normal mitochondrial function is essential for meeting the high energy demands of developing germ cells, while regulating critical processes such as ROS generation, apoptosis, and quality control (9). As autophagy relies on functional mitochondria to provide adenine triphosphate (ATP) for its processes, it must be tightly coordinated with mitochondrial bioenergetics. The adenine nucleotide translocator (ANT) family of ADP/ATP translocases likely links mitochondrial metabolism to autophagic quality control in the testes.

Inflammation is a major cause of male infertility, accounting for about 6-15% of all cases of reproductive problems (10,11). During inflammation, testicular macrophages play an important role in stimulating the production of pro-inflammatory factors. These pro-inflammatory factors not only induce ROS production, but they also disrupt gonadal hormone production and interfere with normal spermatogenic activities (12). For example, interleukin (IL)-6, which is largely released by macrophages, decreases sperm motility and number while increasing malondialdehyde synthesis (13,14). Moreover, increased IL-1β expression is associated with decreased testosterone in stromal cells and decreased spermatogenesis intensity (15). In general, ROS and pro-inflammatory cytokines work synergistically to cause sustained damage to the spermatogenic environment and eventually lead to infertility.

In eukaryotes, ANT acts as a mitochondrial inner protein in the membrane required for oxidative phosphorylation and ATP generation. ANT promotes the movement of adenosine diphosphate (ADP) and ATP through the inner membrane, thereby maintaining cellular energy homeostasis (16). The expression of ANTs varies across tissues, and distinct isoforms play unique roles in biological processes. *Ant1* is mainly expressed in heart and skeletal muscle tissues, whereas *Ant2* is found in a variety of tissues. *Ant3* has widespread expression across several organs, with its transcript abundance being directly correlated with oxidative metabolism. Conversely, *Ant4* is expressed exclusively in the testes, particularly in spermatocytes (17). Recent research has suggested that *Ant* expression may affect diverse biological phenomena. Various diseases, including cancer, neurodegeneration, and congenital muscular dystrophy, have been linked to *Ant* dysfunction. For example, the upregulation of the *Ant* gene by *Rcan1* in Alzheimer's disease increases the ATP-ADP exchange rate, which opens mitochondrial permeability transition pores and boosts cytochrome c (CytC) release, finally causing apoptosis (18). In non-alcoholic fatty liver disease, targeted *Ant2* disruption improves uncoupled respiration and reduces obesity and insulin resistance (19). Previous studies have shown that male mice lacking *Ant4* exhibit early meiotic arrest at the leptotene spermatocyte stage, increased germ cell apoptosis, and complete infertility (20,21). This demonstrates the absolute requirement of *Ant4* for spermatogenesis and reproductive functions. However, the mechanisms through which *Ant4* regulates testicular development and germ cell quality control remain to be fully elucidated.

## Material and Methods

### Animals

All the animals were bred and raised in a controlled environment at the specific pathogen-free (SPF) Laboratory Animal Center of Wenzhou Medical University. *Ant4* heterozygous (*Ant4* ^+/-^) male mice were generated using CRISPR/Cas9 genome editing technology by Saiye (Suzhou) Biotechnology Co., Ltd., China. Adult *Ant4* ^+/-^ male mice were bred with adult *Ant4* ^+/-^ female mice to create *Ant4* homozygous (*Ant4* ^-/-^) male mice. All animal experiments were authorized by the Wenzhou Medical University Ethics Committee. All research was conducted in compliance with the guidelines established by Wenzhou Medical University's Animal Care and Use Committee.

### RNA extraction and quantitative real-time PCR (qRT-PCR)

Mouse tissues were homogenized in 0.5 mL of TRIzol reagent (Thermo Fisher Scientific, USA) using a tissue homogenizer. After adding 0.1 mL of chloroform, the samples were forcefully shaken for 20 s. The samples were then centrifuged for 15 min at 12,000 *g* and 4°C. All of the RNA remained in the aqueous phase. The aqueous phase was moved to a fresh tube, and 0.5 mL of 100% isopropanol was added to precipitate the RNA. The samples were centrifuged at 15,000 *g* for 10 min at 4°C after being incubated at 25°C for 15 min. Next, the RNA pellet was washed with 1 mL of 75% ethanol and centrifuged at 7,500 *g* for 5 min at 4°C. After air-drying, the pellet was resuspended in 20 μL RNase-free water. The RNA concentration was then measured using a NanoDrop spectrophotometer (Thermo Fisher Scientific). The RNA (2 μg) was used to synthesize cDNA using the HiScript II 1st Strand cDNA Synthesis Kit (Vazyme, China). qRT-PCR was performed in duplicate using the SYBR Green PCR Master Mix (Vazyme) on an Applied Biosystems (USA) 7500 Real-Time PCR System. Supplementary Table S1 provides the primers used for the amplification of these genes. The expression of each target gene was normalized to the *β-actin* gene reference and calculated as 2^−ΔΔCT^.

### Western blot

Tissue from the testes was lysed in a mixture containing RIPA lysis buffer (Cell Signaling Technology, USA) and the protease inhibitor phenylmethylsulfonyl fluoride (PMSF, 1 mM, Sangon Biotech, China). The samples were centrifuged at 14,000 *g* for 10 min at 4°C after being incubated on ice for 15 min. Next, 20 μg protein was denatured for 5 min at 95°C. After sodium dodecyl sulfate-polyacrylamide gel electrophoresis (SDS-PAGE), the proteins were transferred onto polyvinylidene difluoride (PVDF) membranes (Bio-Rad, USA). After blocking with 5% milk for 90 min at 25°C, the membranes were incubated with primary and secondary antibodies. Each protein expression was compared to that of β-actin. Finally, the bands were analyzed using ImageJ software (NIH, USA). The primary antibodies used for western blot analysis are listed in Supplementary Table S2. The secondary antibody used was a horseradish peroxidase-conjugated anti-rabbit/mouse IgG (7074/7076, Cell Signaling Technology, 1:2,000).

### Immunohistochemistry

Immunohistochemistry was performed on mouse testis tissue sections using standard protocols: after being dewaxed and rehydrated, the paraffin sections were repaired with sodium citrate repair solution (P0083; Beyotime, China). Next, sections were incubated with anti-LC3B (18725-1-AP, Proteintech, USA; 1:200) and anti-F4/80 (70076, Cell Signaling Technology; 1:200) primary antibodies overnight at 4°C. The slices were then treated with biotinylated secondary antibodies and incubated with peroxidase-linked streptavidin (Nakasugi Golden Bridge, China). Hematoxylin was used as a counterstaining agent; adding 3,3'-diaminobenzidine (DAB) allowed the staining to be visualized with a microscope (Nikon, Japan).

### Mouse fertility test

Eight-week-old sexually mature *Ant4* ^-/-^ male or wild-type male mice were caged with two wild-type female mice for two months. The number of puppies in each litter was recorded. Mean litter size represents the average number of pups from all males tested.

### Sperm count

The sperm count in the caudal epididymis was estimated using the technique reported by Wang (22). The caudal epididymis was dissected and cut into little pieces using scissors. The pieces were then placed in 2 mL of phosphate-buffered saline (PBS) solution and incubated at 37°C for 15 min. After incubation, the diluted sperm suspension was transferred to a hemocytometer and left to stand for 2 min. The sperm count was determined using an electron microscope (Nikon).

### Hematoxylin and eosin staining and histological observation

Tissue samples from the testis and epididymis were set in 4% paraformaldehyde for 24 h and dried in a graduated ethanol series. The samples were then embedded in paraffin and cut into slices of 5-μm thickness. Standard procedures for hematoxylin and eosin (HE) staining were followed. Deparaffinized sections were stained with hematoxylin for 7 min, washed briefly in water, and differentiated in acidic alcohol. The sections were stained with eosin for 10 seconds and then dehydrated using a graded alcohol series. Finally, slides were covered with neutral gum and observed using a light microscope (Nikon).

### Proteomic analysis

Testes were obtained from 10-week-old wild-type (n=3) and *Ant4* knockout (n=3) mice (C57BL/6 background). Proteins were extracted from whole testes by the use of RIPA lysis buffer containing proteinase inhibitors. After quantification using the BCA assay, 200 μg of protein from each sample was digested with trypsin and desalted using reverse-phase solid-phase extraction (SPE). Peptide samples were analyzed in triplicate using a Q Exactive Orbitrap mass spectrometer (USA) coupled with a Dionex UltiMate 3000 UHPLC (NanoLC platform, USA) equipped with trap (C18 PepMap 100, USA) and analytical (Acclaim PepMap 100, USA) columns. The raw MS files were processed using MaxQuant (ver. 1.5.7.4; Germany) and matched against the UniProt Mus musculus database (02/2018; USA). TMT labeling was used to enable the multiplexing of 10 samples in one analysis. Peptide and protein identifications were filtered using a 1% false discovery rate (FDR). Differential expression analysis was performed using the Perseus software (ver. 1.6.1.4; Germany); significant differences were considered as P<0.05 (*t*-test) and abs(log2FC) >0.585 (1.5-fold change). Heatmaps and volcano plots were constructed using the ggplot2 package from the R software (R Core Team).

### Transmission electron microscopy (TEM)

Adult mice were sacrificed for TEM analysis. Their testes were removed and cut into small pieces (1×1 mm), which were then fixed in a solution containing 2.5% glutaraldehyde and 2% paraformaldehyde in 0.1 M PBS for 2-3 h. After being washed with 1xPBS, the samples were post-fixed with 1% osmium tetroxide in 0.1 M PBS (pH 7.4) for 2 h at 4°C. Subsequently, they were dehydrated in a series of graded ethanol solutions (30-100%). Next, the samples were immersed in epoxy resin and sectioned into ultrathin sections (60-80 nm) using an ultramicrotome. The ultrathin slices were contrasted with uranyl acetate and lead citrate and then photographed using a transmission electron microscope (TECNAI G2 F20, Philips, Netherlands) at 120 kV.

### Adenosine triphosphate assay

Testicular tissue was homogenized using an ATP assay lysate (S0026; Beyotime). ATP levels in the tissue were measured using luciferase activity. ATP levels were normalized against protein concentrations determined by a BCA Protein Assay Kit (P0009; Beyotime).

### ROS analysis

ROS analysis was conducted using frozen testicular tissue sections stained with dihydroethidium (DHE), according to the manufacturer's instructions (D7008; Sigma, USA).

### Hydrogen peroxide assay

Hydrogen peroxide concentration was determined using a colorimetric assay kit as directed by the manufacturer (S0038; Beyotime). Hydrogen peroxide oxidizes ferric ions to ferrous ions, which then react with xylenol orange in a specialized solution to form a purple product. H_2_O_2_ concentration was measured at 560 nm after tissue samples were homogenized in lysis buffer.

### Statistical analysis

Data are reported as means±SE. The Shapiro-Wilk test was first utilized to determine normality of data distribution for each experiment. Those meeting normality assumptions were analyzed by Student's *t*-tests for two group comparisons with statistical significance defined as P<0.05. Non-normally distributed data were assessed using the Mann-Whitney U test. One-way ANOVA with *post hoc* Tukey's testing was applied for multi-group comparisons across tissue types, with P<0.05 considered significant.

## Results

### Loss of *Ant4* led to male sterility in mice

**Figure 1:**
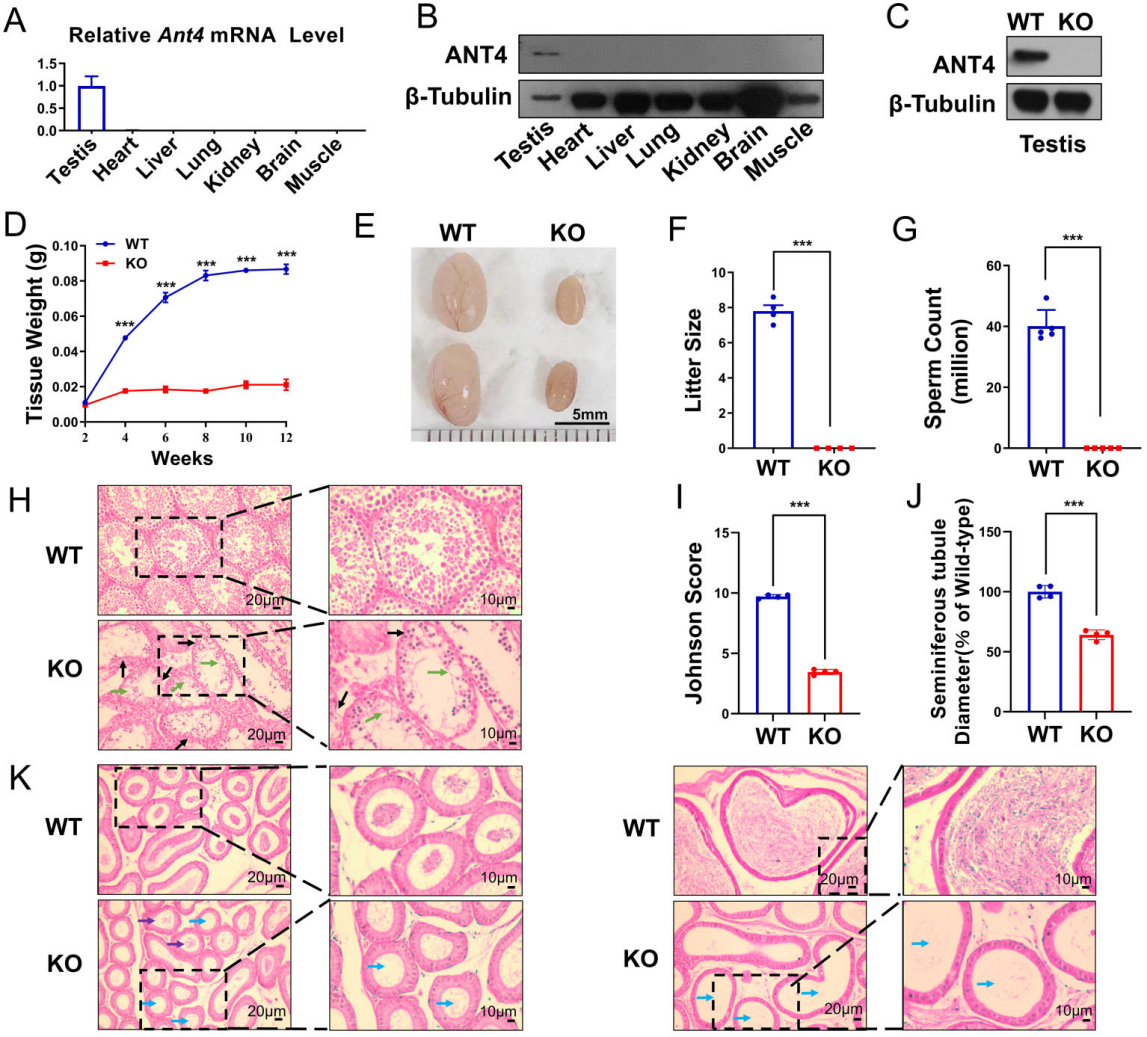
Loss of *Ant4* results in male sterility. **A**, mRNA expression of *Ant4* in different tissues (n=3). **B**, Protein expression of ANT4 in different tissues. **C**, Protein expression of ANT4 in testes of wild type (WT) and Ant4^-/-^ knockout (KO) mice. **D**, Testicular growth curves (n=3). **E**, Testis size. **F**, Average litter size (n=4). **G**, Analysis of sperm count in the cauda epididymis (n=5). **H**, HE staining of testes. Black arrows indicate seminiferous tubular atrophy. Green arrows indicate vacuole-like changes and disintegration of germinal epithelium with a notable reduction in the spermatogenic cell series. **I**, Johnsen score (n=4). **J**, Seminiferous tubule diameter (n=4). **K**, HE staining of caput and cauda epididymidis. Purple arrows indicate sloughed germ cells or cellular debris. Blue arrows indicate signs of apparent sperm stasis (scale bars 20 and 10 μm). Data are reported as means±SE. ***P<0.001 (Student's *t*-test and one-way ANOVA with *post hoc* Tukey's test).

We conducted RT-PCR and western blot analyses to study the expression pattern of *Ant4.* We found that it was exclusively expressed in the testis (Figure 1A and B). We developed an *Ant4* knockout mouse model using CRISPR/Cas9 technology to examine the role of *Ant4* in male reproduction. Western blotting confirmed successful knockout of *Ant4* gene (Figure 1C). Our findings indicated that the testicular weight of *Ant4* ^-/-^ mice was considerably lower than that of wild-type mice after P14, and the testis size of adult *Ant4* ^-/-^ mice was much reduced (Figures 1D and E). Fertility tests showed that *Ant4* ^-/-^ male mice were completely sterile (Figure 1F), and adult *Ant4* ^-/-^ mice had no spermatozoa in their cauda epididymis (Figure 1G). Histological analysis revealed that the size of seminiferous tubules was significantly reduced and the morphology of the epithelium was severely impaired in *Ant4* ^-/-^ mice testes. The spermatogenic epithelium exhibited vacuole-like changes. Furthermore, the seminiferous tubules showed disintegration of germinal epithelium with a notable reduction in the spermatogenic cell series (Figure 1H-J). No mature spermatozoa were observed in both the caput and cauda epididymides (Figure 1K).

### *Ant4* deficiency resulted in oxidative damage

**Figure 2:**
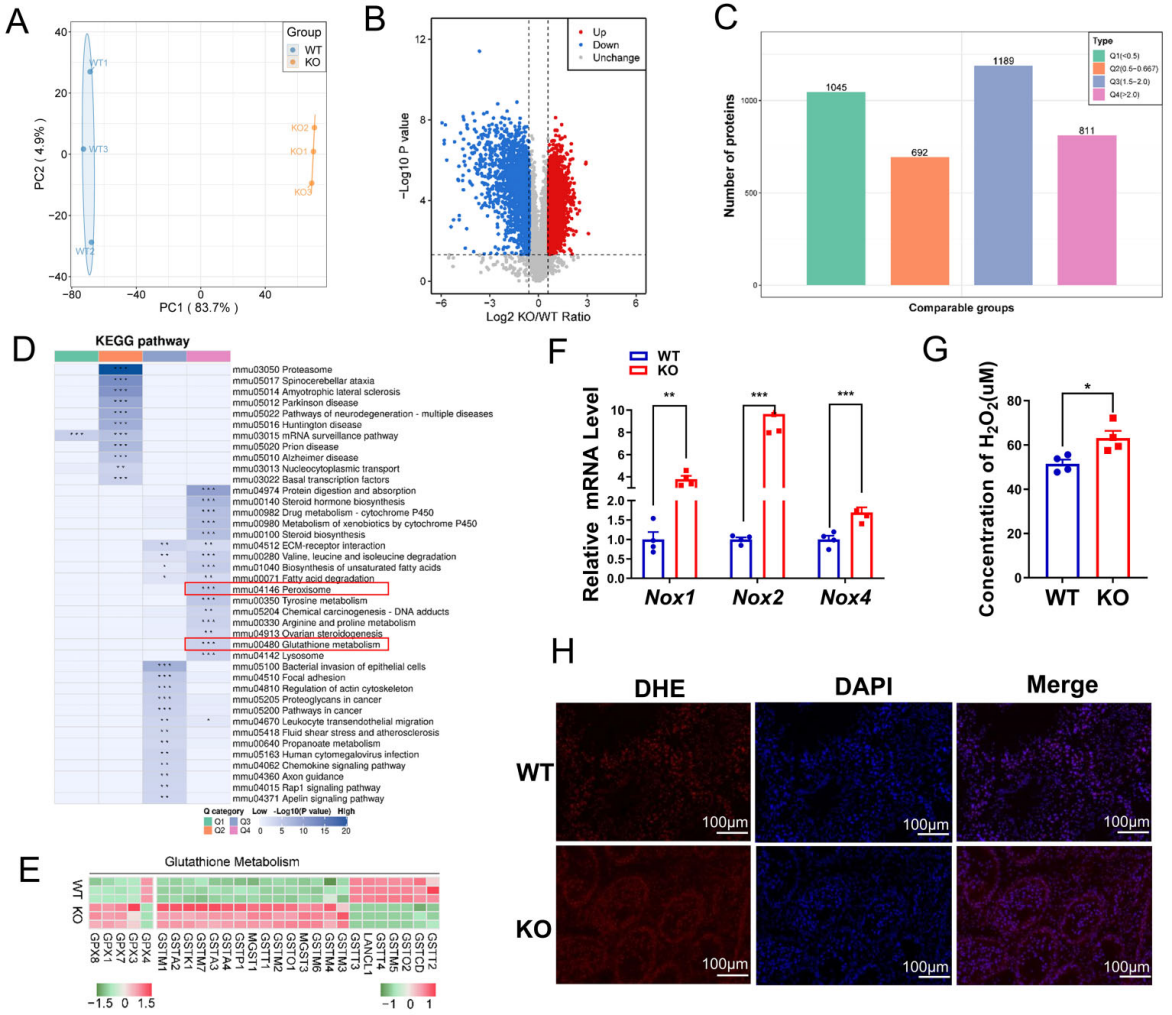
*Ant4* deficiency results in oxidative damage. **A**, Intergroup differences in testes of wild-type (WT) and Ant4^-/-^ knockout (KO) mice. **B**, Volcano graph shows the difference in protein levels in testes of mice (n=3). **C**, The protein was divided into four parts (Q1-Q4) according to different fold changes. **D**, Analysis of total Kyoto Encyclopedia of Genes and Genomes (KEGG) protein enrichment in mice (n=3). **E**, Cluster analysis of glutathione metabolism-related proteins in testis of mice (n=3). **F**, mRNA expression levels of *Nox1*, *Nox2*, and *Nox4* in testes of mice (n=4). **G**, H_2_O_2_ levels in testicular tissue of 10-week-old mice (n=4). **H**, DHE fluorescence (red) in testes of 10-week-old mice (n=3) (scale bars 100 μm). Data are reported as means±SE. *P<0.05, **P<0.01, ***P<0.001 (Student's *t*-test).

We used a proteomics approach to evaluate the impact of *Ant4* knockout on mouse spermatogenesis, and found that the testes of wild-type and *Ant4* ^-/-^ mice showed good reproducibility and significant differences between the two groups (Figure 2A). Compared to wild-type testes, 2,000 upregulated and 1,737 downregulated proteins were detected in the testes of *Ant4* knockout mice (Figure 2B). Next, we compared the functional similarities and differences between proteins of different levels of expression. To achieve this, we categorized the proteins into four groups based on their respective differential expression multiples labeled Q1 to Q4 (Figure 2C). Subsequently, we performed an enrichment analysis using the Kyoto Encyclopedia of Genes and Genomes (KEGG) database for each protein group. Functional clustering analysis was also conducted to group proteins with similar functional properties. We found that proteins in the Q4 group were enriched in many cellular processes such as various amino acid metabolisms, protein digestion, and absorption, which are important for mitochondrial and cellular metabolism (Figure 2D). Two of these particular functions, peroxisome and glutathione metabolism, are closely related to the maintenance of redox balance. Peroxisomes are involved in the production and clearance of ROS (23), whereas glutathione metabolism is vital for cellular antioxidant defense and redox regulation (24). The absence of *Ant4* also significantly altered the expression of genes associated with glutathione metabolism (Figure 2E). These findings strongly suggested that *Ant4* regulated the testicular redox balance, an important aspect of cellular health and function. We used RT-PCR to ascertain the relative mRNA levels of oxidative stress indicators in the testes and to determine whether *Ant4* affected testis oxidation and antioxidant balance. The findings demonstrated that *Nox1*, *Nox2*, and *Nox4* expression levels increased in *Ant4* ^-/-^ mice testes (Figure 2F). Furthermore, H_2_O_2_ and ROS levels (measured using the fluorescent probe DHE) were higher in the testes of *Ant4* ^-/-^ mice than in wild-type mice (Figure 2G and H). Therefore, our findings suggested that the loss of *Ant4* disrupted the balance between oxidation and antioxidation in the testis, leading to oxidative stress.

### Disruption of signaling pathways regulating autophagy upon loss of *Ant4*

**Figure 3:**
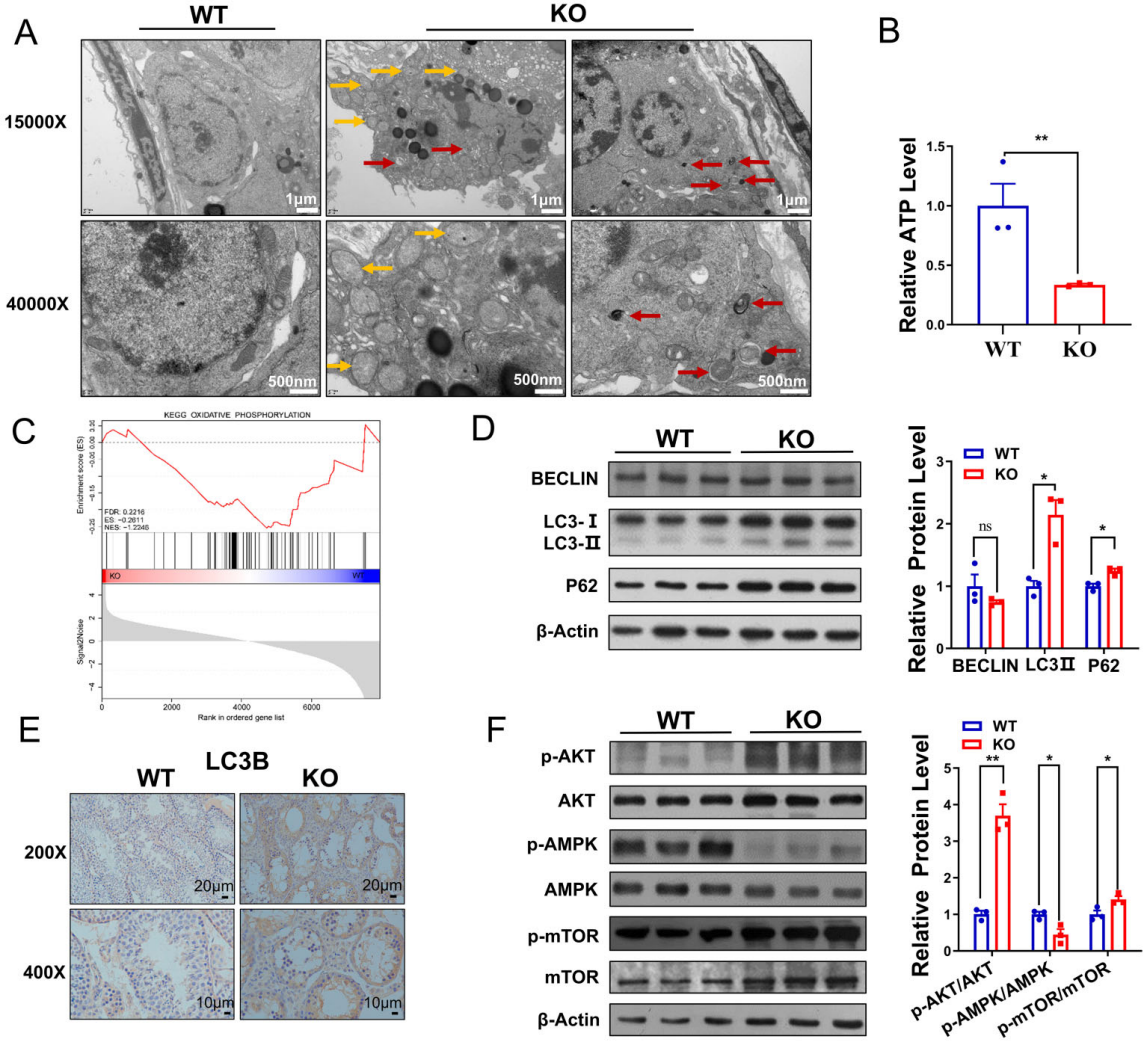
Disruption of signaling pathways regulating autophagy upon loss of *Ant4*. **A**, Transmission electron microscopy images of testes from 10-week-old wild-type (WT) and Ant4^-/-^ knockout (KO) mice. Yellow arrows indicate swelling mitochondria. Red arrows indicate autophagosomes (scale bars 1 μm and 500 nm). **B**, Adenine triphosphate (ATP) levels in testicular tissue of 10-week-old mice (n=4). **C**, Gene set enrichment analysis (GSEA) for OXPHOS. **D**, Western blot images of BECLIN, LC3B (LC3 I and II), and P62 and the statistical results for 10-week-old mice (n=3). **E**, LC3B immunohistochemical staining of testicular sections from 10-week-old mice (n=3) (scale bars 20 and 10 μm). **F**, Western blot images of p-AKT, AKT, p-AMPK, AMPK, p-mTOR, and mTOR, and the ratios of p-AKT and AKT, p-AMPK and AMPK, and p-mTOR and mTOR for 10-week-old mice (n=3). Data are reported as means±SE. *P<0.05, **P<0.01 (Student's *t*-test), ns: non-significant.

We used TEM to investigate the ultrastructural changes in the testes of the *Ant4* knockout mice. TEM analysis showed that wild-type mouse testes exhibited normal ultrastructure, whereas the testes of *Ant4* ^-/-^ mice displayed abnormal mitochondrial architecture, characterized by mitochondrial swelling, loss of cristae, and accumulation of autophagosomes (Figure 3A), suggesting a potential impairment in mitochondrial function. ATP levels in the testes of Ant4^-/-^ mice were also significantly lower than in wild-type mice (Figure 3B). We performed gene set enrichment analysis (GSEA) and found that the OXPHOS signaling pathway was significantly downregulated in *Ant4* ^-/-^ mice (Figure 3C). Autophagy is essential for the elimination of damaged organelles, including mitochondria. Autophagy inhibition often leads to the accumulation of defective mitochondria. To determine whether *Ant4* deletion affected the level of autophagy in the testes of male mice, we monitored the autophagy markers BECLIN, LC3B, and P62. LC3B is commonly used to monitor autophagy and processes associated with autophagy. Deleting *Ant4* enhanced the number of autophagosomes and the level of the autophagy marker LC3 II (Figure 3D). Immunohistochemistry also indicated enhanced LC3B staining in the testes of *Ant4* ^-/-^ mice (Figure 3E). While BECLIN, which initiates autophagosome formation, did not change significantly, P62, a protein degraded by autophagy, was found to increase in the testis of *Ant4* ^-/-^ mice (Figure 3D). Thus, our results suggested that deletion of the *Ant4* gene led to defective autophagy in the testes of male mice, resulting from impaired autophagic degradation rather than formation. Furthermore, *Ant4* ^-/-^ mice had much higher levels of p-AKT and p-mTOR (negative regulators of autophagy), whereas p-AMPK, a positive regulator of autophagy, was notably lower (Figure 3F). These findings suggested that *Ant4* knockdown may inhibit autophagy by affecting the AKT-AMPK-MTOR pathway.

### Effect of *Ant4* knockout on testicular inflammation and apoptosis

**Figure 4:**
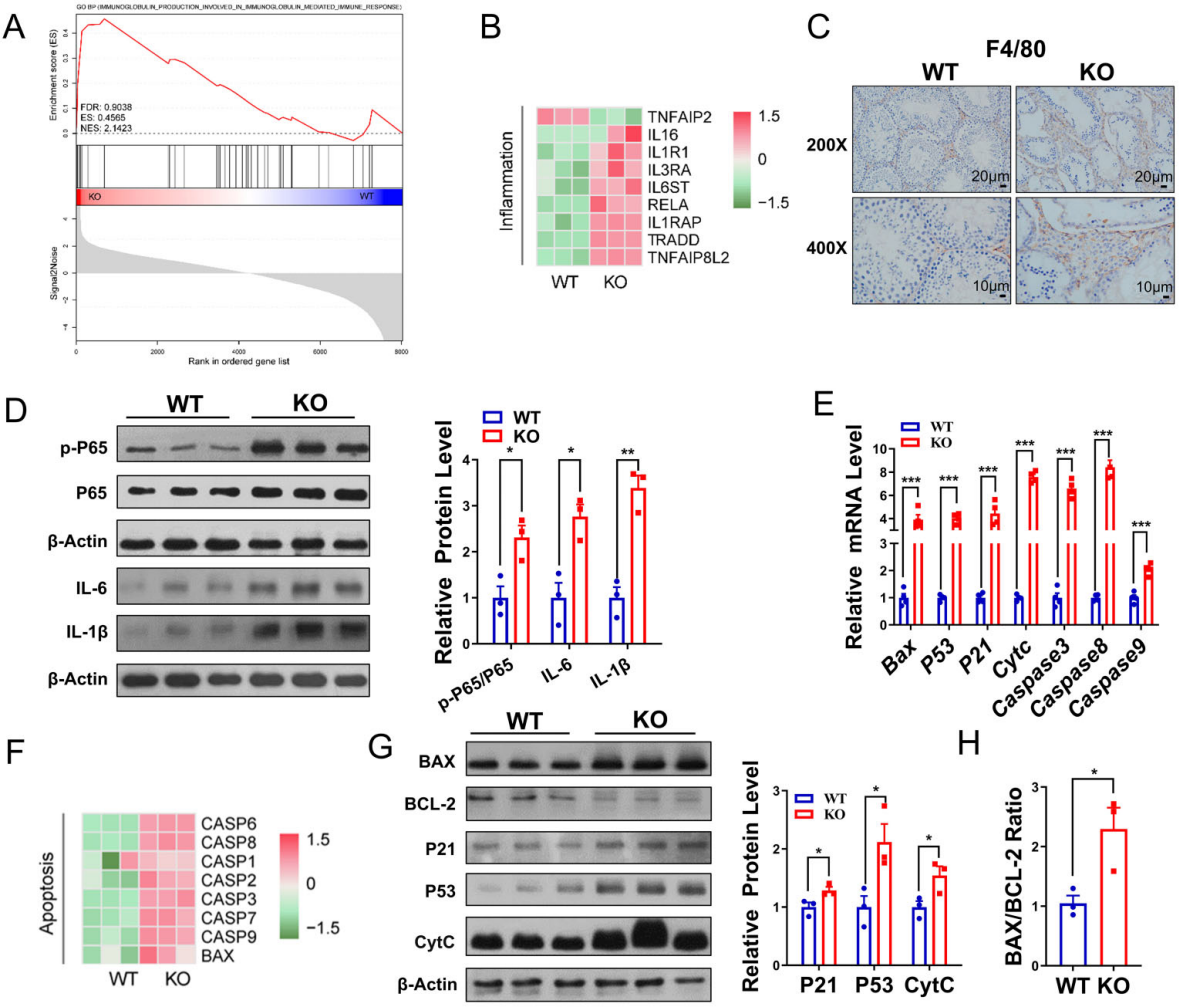
Effect of *Ant4* knockout (KO) on testicular inflammation and apoptosis. **A**, Gene set enrichment analysis (GSEA) for immune response. **B**, Cluster analysis of inflammation-related proteins in the testis of wild-type (WT) and KO mice (n=3). **C**, F4/80 immunohistochemical staining of testicular sections from mice (n=3). Scale bars 20 and 10 μm. **D**, Western blot images of P65, p-P65, interleukin (IL)-6, and IL-1β and statistical results for mice (n=3). **E**, mRNA expression levels of apoptosis-related genes in testes of mice (n=4). **F**, Cluster analysis of apoptosis-related proteins in testis of mice (n=3). **G**, Western blot images of BAX, BCL-2, P21, P53, and CytC and the statistical results (n=3). **H**, The ratio of BAX to BCL-2 (n=3). Data are reported as means±SE. *P<0.05, **P<0.01, ***P<0.001 (Student's *t*-test).

Numerous studies have demonstrated that inflammation plays a significant role in developing male infertility. Autophagy and oxidative stress are strongly related to cell death and are central to the progression of several inflammatory diseases (25,26). GESA analysis showed that the immune response pathway was significantly upregulated in *Ant4* ^-/-^ mice (Figure 4A). In addition, the proteins associated with inflammation showed an increasing trend in the heat map (Figure 4B). We measured F4/80-labeled macrophages to assess the effect of *Ant4* knockout on testicular inflammation. The number of F4/80 positive cells was significantly higher in *Ant4* ^-/-^ testes compared to wild-type mice (Figure 4C), indicating increased recruitment of inflammatory cells following *Ant4* deletion. The nuclear factor-kappa B (NF-kB) signaling pathway is known to regulate inflammatory responses; p65 is an essential member of the NF-kB family, and its activation triggers the release of many inflammatory factors (27). After *Ant4* deletion, NF-kB signaling proteins P65 and p-P65 levels increased. The ratio of p-p65/p65 in the testes of *Ant4* ^-/-^ mice was also significantly higher than that of wild-type mice (Figure 4D). There was also a significant increase in the levels of pro-inflammatory cytokines such as interleukin (IL)-6 and IL-1β (Figure 4D). Our data suggested that the loss of *Ant4* can enhance inflammation by activating the NF-kB signaling pathway. Researchers have found that deletion of the *Ant4* gene can trigger apoptosis in mouse testes (20). Consistent with this, the mRNA expression of apoptosis-related genes, such as *Bax*, *P53*, *P21*, *Cytc*, *Caspase-3*, *-8*, and *-9* was considerably elevated in the testes of Ant4^-/-^ mice compared to wild-type mice (Figure 4E). Apoptosis-related proteins also showed an increasing trend in Ant4^-/-^ mouse testis (Figure 4F). Additionally, the lack of *Ant4* resulted in the upregulation of pro-apoptotic proteins, including BAX, P53, P21, and CytC, and downregulation of the anti-apoptotic protein BCL-2 and a reduction in the BCL-2/BAX protein ratio (Figure 4G and H). Our results suggested that the loss of *Ant4* affects male fertility by promoting inflammation and apoptosis.

## Discussion

In this study, we used 10-week-old mice because both male and female mice at this stage are considered young adults that have reached sexual maturity, and thus, the effect of *Ant4* deficiency on male reproduction can be more intuitively demonstrated. We found that *Ant4* deficiency disrupted the redox balance, while high levels of ROS can cause male infertility via a variety of pathways. Simultaneously, *Ant4* loss can reduce OXPHOS, ATP, and autophagy levels, which may affect the ability to meet energy demands during spermatogenesis. In addition, *Ant4* deficiency can lead to increased testicular inflammation and apoptosis, which can cause damage to germ cells. Our results showed that *Ant4* is important for maintaining testicular homeostasis, suggesting that *Ant4* regulates the general turnover of cellular contents necessary for germ cell health.

Existing evidence indicates that ROS is a key factor in healthy sperm development and maintenance of normal reproductive capacity. High ROS levels can cause sperm DNA fragmentation, lipid peroxidation, apoptosis, and protein damage (12). NADPH oxidases (NOX) are electron-transporting membrane enzymes whose primary purpose is to produce ROS (28). The expression levels of *Nox1*, *Nox2*, and *Nox4* can be used to evaluate the oxidative stress level of mouse testes (29). To determine whether the deletion of *Ant4* causes oxidative stress, we measured the relative mRNA levels of prooxidative stress genes (*Nox1*, *Nox2*, and *Nox4*) in testis and found that their expression was increased. Moreover, the levels of ROS and H_2_O_2_ in the testes also increased, indicating that the testes were in a state in which the oxidative capacity exceeded the antioxidant capacity. Thus, the imbalance between testicular oxidation and antioxidation caused by *Ant4* deficiency may be a key factor leading to male infertility.

Normal mitochondrial function is critical for mammalian spermatogenesis as it is involved in energy generation, maintenance of redox homeostasis, and apoptotic pathways. The impaired mitochondrial function increases the likelihood of apoptosis by impairing oxidative phosphorylation or generating excess ROS (30). In turn, high levels of ROS can damage the mitochondria, leading to a vicious cycle in germ cells. The present study found that the loss of *Ant4* impaired mitochondrial morphology and functionality, reduced ATP levels, and disturbed OXPHOS, underscoring the critical role of *Ant4* in maintaining mitochondrial homeostasis. Additionally, previous studies demonstrated that autophagy is closely related to male reproduction, and impaired autophagy can decrease sperm count and motility, inhibit testosterone synthesis, affect sexual behavior, and reduce fertility (8,31). Autophagy dysfunction leads to an accumulation of damaged mitochondria, which increases the generation of ROS and worsens testicular injury. Knockdown of *Ant4* causes autophagosome accumulation in mice testes. Furthermore, the study detected the autophagy-related proteins BECLIN, LC-3 II, and P62 and found that loss of *Ant4* inhibits autophagic degradation (32,33). Moreover, *Ant4* loss lowered AMPK phosphorylation while increasing MTOR and AKT phosphorylation, suggesting that autophagic flux and the AKT-AMPK-MTOR signaling pathway were affected in *Ant4*-deficient mice.

Inflammation of the male reproductive tract is implicated in infertility, involving the activation of signaling pathways such as NF-κB (34). The phosphorylated NF-κB p65 subunit marks pathway activation and regulates pro-inflammatory cytokines (35,36). *Ant4* deficiency increased macrophage infiltration, activated NF-κB signaling, and upregulated pro-inflammatory cytokines, suggesting *Ant4* affects fertility by promoting inflammation. Apoptosis, programmed cell death induced by inflammation and ROS, can impair spermatogenesis by eliminating germ cells (37,38). The mitochondrial-dependent apoptotic pathway releases CytC, which triggers cell death (39,40). We showed that *Ant4* deficiency decreased anti-apoptotic BCL2 but increased pro-apoptotic BAX, p21, p53, and CytC, indicating that *Ant4* loss promoted testicular apoptosis, likely due to mitochondrial damage, oxidative stress, and inflammation.

Our study is the first to systematically analyze how *Ant4* orchestrates testicular homeostasis with quality control to support germ cell development. By coordinating these mechanisms, *Ant4* helps to generate an optimized environment for spermatogenesis where germ cells can differentiate and survive. In males lacking *Ant4*, the loss of this homeostatic balance across interconnected pathways leads to disruption of the testicular niche, cell damage, and death, culminating in impaired reproductive function. Continued exploration of *Ant4* regulation may provide new treatment opportunities for male infertility arising from mitochondrial dysfunction or insufficient organelle turnover. Diagnosing and correcting aberrations within this network can provide effective support for normal spermatogenesis. Our study established that *Ant4* is essential for uniting cellular energetics, protein quality control, and redox homeostasis in the testes, providing foundational insights into how naturally balancing these processes enables germ cell health and fertility.

## Supplementary Material

Click here to view [pdf].
