# Supplementary material for: The essential role of adenine nucleotide translocase 4 on male reproductive function in mice
Source: Braz J Med Biol Res. 2024 May 20;57:e13590. doi: 10.1590/1414-431X2024e13590 (PMC11136480; doi:10.1590/1414-431X2024e13590)
Supplement: Supplementary file 1 [file 1414-431X-bjmbr-57-e13590-suppl.pdf]

Table S1. Primer sequences used in this study.

Application of primers	Sequences
Ant4 KO genotyping	F 5'-CTAGAGGCCGTGATGGGAATTTTG-3'<br>R 5'-ATTGCACTTTGCTAAGTCCCATTCAC-3'<br>He/Wt-F 5'-GGCTACAAGTTGGCATAATGAAGTAGA-3'
RT-PCR primer of *Ant4*	F 5'-GCTGGAGCAACATCCTTGTGT -3'<br>R 5'-GTCACCCAAACCCGTGAACT -3'
RT-PCR primer of *Nox1*	F 5'-TCCTTCGCTTTTATCGCTCC-3'<br>R 5'-TCGCTTCCTCATCTGCAATTC-3'
RT-PCR primer of *Nox2*	F 5'-TCCTATGTTCCTGTACCTTTGTG -3'<br>R 5'-GTCCCACCTCCATCTTGAATC-3'
RT-PCR primer of *Nox4*	F 5'-TCCAAGCTCATTTCCCACAG-3'<br>R 5'-CGGAGTTCCATTACATCAGAGG-3'
RT-PCR primer of *Bax*	F 5'-CCGGCGAATTGGAGATGAACT -3'<br>R 5'-CCAGCCCATGATGGTTCTGAT -3'
RT-PCR primer of *P53*	F 5'-GTCACAGCACATGACGGAGG -3'<br>R 5'-TCTTCCAGATGCTCGGGATAC -3'
RT-PCR primer of *P21*	F 5'-CCTGGTGATGTCCGACCTG -3'<br>R 5'-CCATGAGCGCATCGCAATC -3'
RT-PCR primer of *Cytc*	F 5'-TGAGCGAGTACAACAAGGGC -3'<br>R 5'-GGCTGGTCATGGAAAGGACAG -3'
RT-PCR primer of *Caspase3*	F 5'-TGGTGATGAAGGGGTCATTTATG -3'<br>R 5'-TTCGGCTTTCCAGTCAGACTC -3'
RT-PCR primer of *Caspase8*	F 5'-TGCTTGGACTACATCCCACAC -3'<br>R 5'-TGCAGTCTAGGAAGTTGACCA -3'
RT-PCR primer of *Caspase9*	F 5'-TCCTGGTACATCGAGACCTTG -3'<br>R 5'-AAGTCCCTTTCGCAGAAACAG -3'

Table S2. Primary antibodies used for western blot analysis in this study.

Protein name	Manufacturer (catalogue number)	Applications (working dilution)
ANT4	Biorbyt (orb215309)	1:1000
β-TUBLIN	Santa Cruz Blotechnology (5274)	1:5000
LC3B	Proteintech (18725-1-AP)	1:1000
p62	Cell Signaling Technology (39749S)	1:1000
BECLIN	Proteintech (66665-1-Ig)	1:2000
β-ACTIN	Proteintech (20536-1-AP)	1:2000
AKT	Cell Signaling Technology (2938)	1:1000
p-AKT	Cell Signaling Technology (12694)	1:1000
AMPK	Cell Signaling Technology (5832)	1:1000
p-AMPK	Cell Signaling Technology (2535)	1:1000
mTOR	Cell Signaling Technology (2972S)	1:1000
p-mTOR	Cell Signaling Technology (2971S)	1:1000
P65	Cell Signaling Technology (8242)	1:1000
p-P65	Cell Signaling Technology (3033)	1:1000
IL-6	Cell Signaling Technology (12912)	1:1000
IL-1β	Cell Signaling Technology (12242)	1:1000
BAX	Proteintech (60267-1-Ig)	1:5000
BCL-2	HuaBio (ET1603-11)	1:1000
p53	Cell Signaling Technology (2524S)	1:1000
p21	Proteintech (10355-1-AP)	1:1000
Cytochrome C	Proteintech (10993-1-AP)	1:8000
